# Polygenic scores for low lung function and the future risk of adverse health outcomes

**DOI:** 10.1186/s12933-022-01661-y

**Published:** 2022-11-03

**Authors:** Suneela Zaigham, Isabel Gonçalves, Regeneron Genetics Center, Gunnar Engström, Jiangming Sun

**Affiliations:** 1 Department of Clinical Sciences Malmö, Lund University, Malmo, Sweden; 2 Department of Medical Sciences, Uppsala University, Uppsala, Sweden; 3 Department of Cardiology, Skåne University Hospital, Malmo, Sweden; 4 Regeneron Genetics Center, Regeneron Pharmaceuticals, Tarrytown, NY USA

**Keywords:** Genetic risk score, Lung function, Diabetes, Risk prediction

## Abstract

**Aims:**

Reduced lung function and adverse health outcomes are often observed. This study characterizes genetic susceptibility for reduced lung function and risk of developing a range of adverse health outcomes.

**Methods:**

We studied 27,438 middle-aged adults from the Malmö Diet and Cancer study (MDCS), followed up to 28.8 years. Trait-specific Polygenic scores (PGS) for forced expiratory volume in 1 s (FEV_1_) and forced vital capacity (FVC) were constructed for each participant using MDCS genetic data and summary statistics from the latest GWAS of lung function. Linear regression models and cox proportional hazards regression models were used to assess associations between adverse health outcomes and lung function-PGS.

**Results:**

FEV_1_-PGS and FVC-PGS were significantly associated with mean sBP at baseline after adjustments (FEV_1_-PGS Q1 (highest PGS = highest lung function): 140.7mmHg vs. Q4: 141.5mmHg, p-value 0.008). A low FVC-PGS was significantly associated with the risk of future diabetic events after adjustments (Q4 vs. Q1 HR: 1.22 (CI 1.12–1.32), p-trend < 0.001) and had added value to risk prediction models for diabetes. Low FEV_1_-PGS was significantly associated with future coronary events (Q4 vs. Q1 HR: 1.13 (CI: 1.04–1.22), p-trend 0.008). No significant association was found between PGS and sudden cardiac death, chronic kidney disease or all-cause mortality. Results remained largely unchanged in a subgroup of subjects when further adjusted for apolipoproteins.

**Conclusion:**

Genetic susceptibility for reduced lung function is associated with higher sBP, increased risk of diabetes and to a lesser extent, future coronary events, suggesting etiological roles of lung function on these outcomes. Using PGS, high-risk groups could be early detected to implement early lifestyle changes to mitigate the risk.

**Supplementary Information:**

The online version contains supplementary material available at 10.1186/s12933-022-01661-y.

## Introduction

Many observational studies have shown associations between low levels of lung function and future adverse health events, including diabetes, myocardial infarction (MI), sudden cardiac death (SCD) and all-cause mortality [1–5]. Although these relationships have been found independently of commonly shared risk factors (e.g. in cohorts of life-long never smokers), they remain vulnerable to unmeasured confounders present in life-style choices that are difficult to fully take into account in observational studies.

Polygenic scores (PGS) are a useful instrument in providing genetic risk prediction by aggregating the effects from a large number of genetic variants associated with particular traits identified from genome-wide association studies (GWAS) [6]. PGS, being less likely affected by non-genetic factors, have shown promising applications in disease prediction and early screening [7, 8]. PGS were also used to examine putative causal relationships with good statistical power [9] and were suggested as an approach in complement to Mendelian randomization (MR) [9, 10] though horizontal pleiotropy need be well considered. Moreover, PGS, representing a personalized score for evaluating one’s life-time risk, is an emerging approach in precision medicine.

A GWAS has discovered 279 risk loci for chronic obstructive pulmonary disease (COPD) [11]. Based on this, PGS have been constructed in predicting pulmonary diseases such as COPD [12–16]. Less is known for PGS cross trait prediction such as cardio-metabolic events. Although previous MR studies have found lung function to be inversely related to coronary artery disease (CAD) [17–19], stroke, type 2 diabetes mellitus (T2DM) and systolic blood pressure (sBP) [18], we are not aware of studies of PGS for lung function systematically assessing its predictive values in predicting a range of future incident outcomes.

Herein, we assessed the risk of cardio-metabolic outcomes (major adverse cardiovascular events (MACE), sudden cardiac death (SCD), sBP and diabetes), chronic kidney disease (CKD) and all-cause mortality in relation to PGS for forced expiratory volume in 1 s (FEV_1_) and for forced vital capacity (FVC) using the the Malmö Diet and Cancer study (MDCS) in 27,438 subjects.

## Methods

### Study population

**Fig. 1:**
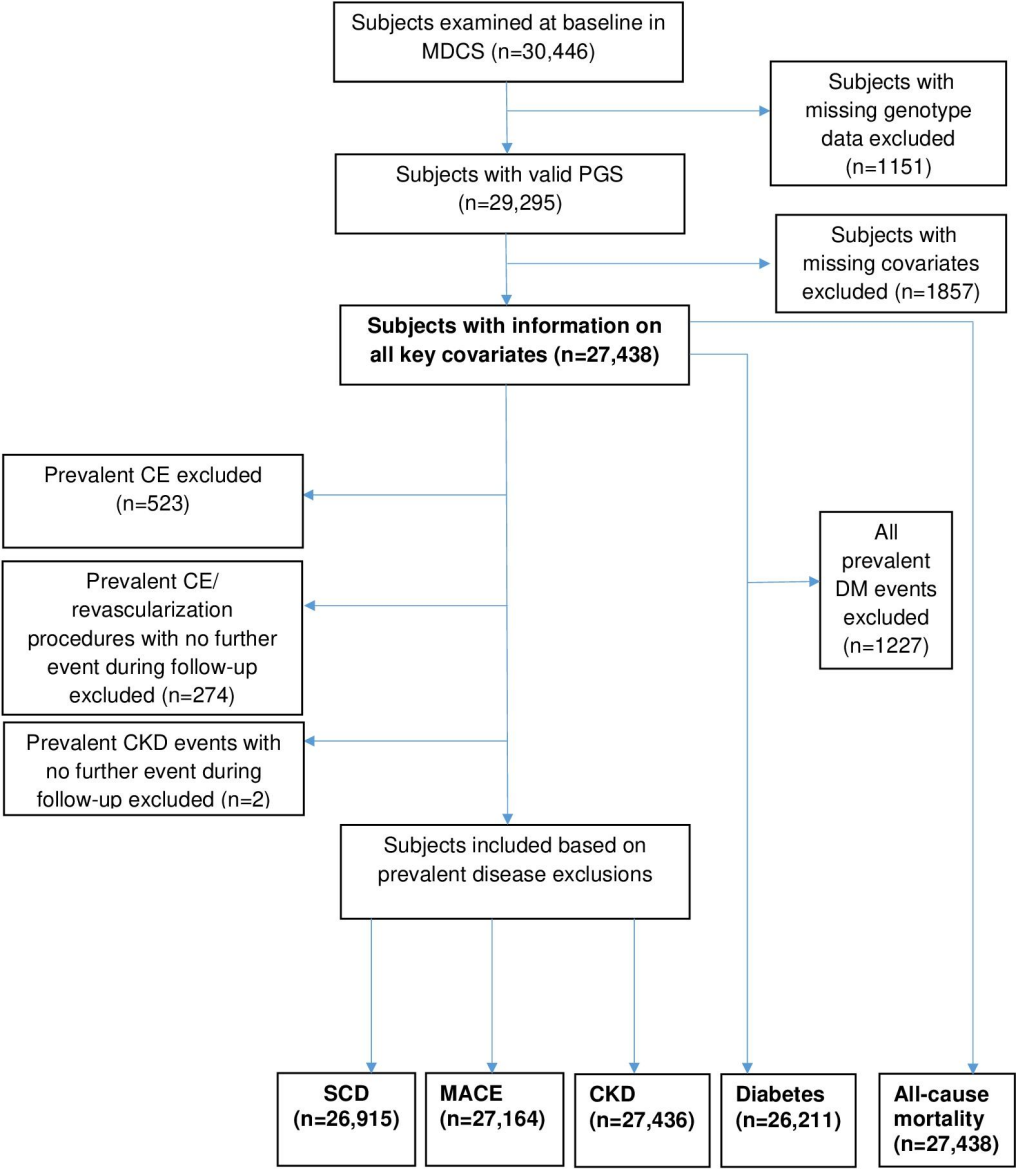
Flow of subjects through the study

The Malmö Diet and Cancer study (MDCS) is a large prospective cohort study of men and women from Malmö, Sweden. Between 1991 and 1996, subjects aged 44–73 years living in Malmö were recruited to take part in the study. All men born between 1923 and 1945 and women born between 1923 and 1950 were invited to participate. Participation rate was approximately 40%. A total of 30,446 men and women underwent baseline examinations. All participants provided written consent, and the Ethical Committee at Lund University, Lund, Sweden approved the study (LU 51–90, LU 2009/633, LU 2011/356). Subjects with missing genotype data were excluded (n = 1151). Additionally, subjects with missing information on key variables for model adjustments were excluded (n = 1857). As exclusion of prevalent disease at baseline is not necessary for prediction of incident events if genetic risk is the exposure, we included prevalent cases where later a further event was recorded for the same subject for coronary events (CE) and chronic kidney disease endpoints but not SCD and diabetes. As SCD by definition is a sudden event in someone who is not known to previously have coronary heart disease, we excluded all prevalent cases of CE for the outcome of SCD. Prevalent cases of diabetes at baseline that occurred earlier than baseline were excluded for the purposes of this analysis to ensure that the majority of diabetes events included were type 2 diabetes mellitus (T2DM). The number of subjects examined for each outcome are illustrated in Fig. 1.

### Baseline examinations

Standing height was measured using a fixed stadiometer calibrated in centimetres. Weight was measured whilst subjects wore light clothing and no shoes using a balance beam scale to the nearest 0.1 kg. Body mass index (BMI) was calculated as weight (kg)/height^2^(m). Blood pressure was measured using a mercury column sphygmomanometer after 10 min of rest in the supine position. Non-fasting blood samples were taking at enrolment. Serum and plasma were separated within one hour and stored at -80 degrees centigrade. Serum concentrations of Apolipoprotein A1 (Apo A-1) and Apolipoprotein B (Apo B) were measured by Quest Diagnostics (*San Juan Capistrano, CA*) using an immunonephelometric assay run on the Siemens BNII (*Siemens, Newark, DE*). Smoking status was ascertained from self-administered questionnaire responses. Higher education was defined as gaining university level education. Low physical activity was defined as the lowest quintile of the physical activity score, described in detail elsewhere [20]. Use of inhaled corticosteroids was defined as R03BA codes in the ATC (Anatomical, Therapeutic, Chemical classification) register. Baseline prevalent diabetes was defined as self-reported history of physician’s diagnosis of diabetes or use of anti-diabetic medication. Baseline prevalent CE and CKD were defined using national registers. No lung function was measured at the baseline MDCS but a subset of participants were re-examined as part of the Malmö Preventive Project cohort (MPP), where spirometry was measured. (n = 14 961 with FEV_1_ (L) and 14 979 with FVC (L)).

### Genotyping and quality control of the Malmö Diet and Cancer study

MDCS participants were genotyped using Illumina GSA v1 genotyping array. Quality controls (QC) on the genotyped variants were conducted by removing variants for probe to genome mismatch, incorrect assignment of allelic variant, failed genotype calling, call rate less than 99%, minor allele frequency (MAF) less than 0.01 or failed Hardy-Weinberg Equilibrium test at p < 1 × 10^− 15^. For sample QC, samples were excluded if genetic sex did not match pedigree sex or had an overall sample call rate less than 90%. The FlashPCA [21] was implemented for principal component analysis (PCA) on the genotyped data. Imputation was performed using the reference panel of Haplotype Reference Consortium (HRC r1.1) [22].

### Polygenic risk scores (PGS) for lung function

A Bayesian regression framework PRS-continuous shrinkage (PRS-CS) [23] was applied to build PGS for lung function. PRS-CS had shown superior performances in the prediction of common diseases and quantitative traits [23]. PRS-CS was used to infer posterior single-nucleotide polymorphism (SNP) effect sizes based on linkage disequilibrium reference panel of 1000 Genomes Project (European sample, n = 503) and GWAS summary statistics on FEV_1_ (n = 400 102) and FVC (n = 400 102) from meta-analysis on the UK Biobank and SpiroMeta, respectively, where age, age squared, sex, height and smoking was adjusted in the association analyses [11]. We restricted SNPs that are biallelic, having MAF no less than 0.01 and consistent directions in sub studies of GWAS of meta-analysis. Using the PLINK (version 1.90) [24], PGS for FEV_1_ and FVC on 29 295 MDC samples were then constructed by aggregating the obtained posterior effect sizes for FEV_1_ (635 689 SNPs) and FVC (634 755 SNPs).

Through the study, PGS for FEV_1_ or FVC are a relative measure of lung function in arbitrary units. High PGS represent high measures of FEV_1_ or FVC, implying high lung function and vice versa.

### Endpoint ascertainment

SCD was defined as a fatal CE where death took place within the first 24 h, in individuals without a previous CE. This included those cases where death occurred outside of hospital. International Classification of Diseases (ICD) 9 codes included 410, 412 and 414; and ICD 10 codes I21, I24 and I25 [25, 26]. Major adverse cardiovascular events (MACE) were defined as CE, coronary artery bypass graft (CABG) or percutaneous coronary intervention (PCI) with ICD 9 codes 410, 412, 414 and ICD 10 codes I20, I21, I24 and I25. For CE, data linkage with the National Cause of Death Registry, Swedish Hospital Discharge registry and the Malmö Myocardial Infarction Register was used to retrieve cases [27]. Incident diabetes was defined using the Malmö HbA1c register (MHR), the Swedish national Diabetes register (NDR), the Swedish hospital discharge register, the Swedish outpatient register, the cause of death register, the Swedish drug prescription register, the regional Diabetes 2000 register of the Scania region and the All New Diabetics in Scania (ANDIS) registry. Incident cases of diabetes were also retrieved from re-examination of MDCS subjects in the MPP (2002–2006), MDCS baseline screening in the cardiovascular cohort (1992–1994), MDCS 5 year rescreening (1997–2001) and MDC cardiovascular rescreening (2007–2012). Incident CKD events were defined as ICD-10 codes N18 or N19. The Swedish inpatient registry had been in operation during the entire follow-up period and data from this registry has been found to have acceptable validity for epidemiological research [28]. All subjects were followed from the baseline examinations until the event of interest, death from other causes, emigration or last follow-up date (31st December 2019), whichever came first.

### Statistical analysis

All statistical analyses were carried out using SPSS V.26 (IBM, Armonk, New York, USA) and STATA v14.0. Cox regression models were implemented to obtain hazard ratios (HR) for all endpoints by quartiles of FEV_1_-PGS and FVC-PGS (Q1 highest PGS –reference) and by 1-SD increase of FEV_1_-PGS and FVC-PGS. Adjustments were kept to a minimum but were made for potentially key confounders known from the literature (Model 1: unadjusted, Model 2: age, sex, height, weight, smoking, sBP, prevalent diabetes (except for the outcome of diabetes), and the first 5 principal components (PCs) of population structures. The outcomes of diabetes and hypertension were additionally adjusted for inhaled corticosteroids. PC allow us to adjust for underlying population structures, where genetic ancestry may explain associations between variants and a specific phenotype. As Apo A-1 and Apo B levels were available for fewer subjects, a further analysis was carried in these subjects after adjustment for Apo A-1 and Apo B (number of subjects by outcome with apolipoproteins available= SCD = 26,035, MACE = 26,273, diabetes = 25,376, CKD = 26,534, all-cause mortality = 26,536 and sBP = 26,536). Time dependent covariate analysis and Kaplan Meier curves were used to assess the proportional hazards assumptions. Using the time-dependent covariate analysis, proportional hazards assumptions were fulfilled for all adjusted outcomes. Univariate regression models were used to assess mean sBP by quartiles of FEV_1_-PGS and quartiles of FVC-PGS, adjusted for potential confounders known from the literature. A p-value of < 0.05 was considered statistically significant, however, to take into account the role of multiple testing, a Bonferroni adjusted p-value of < 0.01 was used to assess p-trend across quartiles.

We assessed the improvement of prediction models of coronary heart disease and diabetes after the addition of PGS for lung function to the prediction models. Performance of the models was assessed using log-likelihood ratio tests and a category-free net reclassification index (NRI) [29]. For incident MACE, risk prediction was carried out in non-diabetic (at baseline) subjects, with no known intermittent claudication and no known previous CE (n = 22,065 subjects). Covariates in the risk prediction model included age, sex, smoking status, sBP, ApoA1 and ApoB and BP medication. For incident diabetes, risk prediction was carried out in non-diabetic subjects (at baseline) (n = 25,655 subjects). Covariates in the risk prediction model included age, sex, sBP, parental history of diabetes, ApoA1, ApoB and BMI.

## Results

Subjects were followed up for an average of 28.8 years (SD ± 6.5). Baseline characteristics are shown in Table 1 for quartiles of FVC-PGS and Supplement Table 1 for quartiles of FEV_1_-PGS.

**Table 1:** Baseline association between risk factors and FVC-PGS (n = 27 438)

	Q1 (highest) − 0.27 to 0.53 (n = 6859)	Q2 − 0.42 to − 0.27 (n = 6860)	Q3 − 0.57 to − 0.42 (n = 6860)	Q4 (lowest) − 1.36 to − 0.57 (n = 6859)	p-value
Age (years)	58.0 (± 7.6)	58.2 (± 7.6)	58.1 (± 7.7)	58.1 (± 7.7)	0.550
Height (cm)	168.4 (± 8.9)	168.6 (± 8.8)	168.7 (± 8.8)	168.8 (± 8.9)	0.020
BMI (kg/m^2^)	25.6 (± 3.9)	25.8 (± 4.0)	25.8 (± 4.0)	25.9 (± 4.0)	< 0.001
Systolic BP (mmHg)	140.5 (± 20.0)	141.4 (± 20.0)	141.1 (± 19.9)	141.8 (± 20.1)	0.001
Current smokers (%)	27.8	28.1	28.6	28.1	0.149
ApoA1*	156.9 (± 27.5)	157.0 (± 28.2)	156.6 (± 28.5)	156.6 (± 28.6)	0.421
ApoB*	106.9 (± 26.0)	106.5 (± 26.5)	107.2 (± 26.2)	107.6 (± 25.5)	0.050
Prevalent CE (%)	1.8	1.9	1.9	2.1	0.278
Prevalent DM (%)	3.7	4.3	4.3	5.6	< 0.001
Inhaled corticosteroid use (%)	1.9	2.1	2.1	2.2	0.251
Higher education (%)	15.2	14.4	14.1	13.2	0.001
Low physical activity (%)**	19.7	19.9	20.8	20.3	0.241

*Data from 26 536 subjects on lipoproteins

** Data from 27,237 subjects on physical activity score

There was a significant association between FEV_1_-PGS and FVC-PGS quartiles and height, sBP and proportion of subjects with prevalent diabetes and proportion with higher education at baseline. Additionally, FVC-PGS quartiles were also significantly associated with BMI (Table 1), whilst FEV_1_-PGS quartiles were significantly associated with inhaled corticosteroid use (**Supplement** Table 1) Of the 27,438 subjects in the baseline MDCS cohort, 14 961 subjects had information on FEV_1_ (L) and 14 979 subjects had information on FVC (L). In these subjects Pearson’s correlation coefficient between FEV_1_-PGS and FEV_1_ (L) was 0.031 (p-value < 0.001) and FVC-PGS and FVC (L) was 0.019 (p-value 0.018). In 27,438 subjects, there was a negative correlation between FEV_1_-PGS and FVC-PGS with height (-0.030, p-value < 0.001 and − 0.018, p-value 0.003, respectively), however height was already adjusted in the GWAS analyses of lung function which may have resulted in over-adjustment for height in the present cohort and therefore observed negative correlations. FEV_1_-PGS was significantly associated with mean sBP at baseline in 27,438 subjects after adjustment for potential confounders (Mean BP (mmHg) FEV_1_-PGS Q1: 140.7 vs. Q4: 141.5, p-value 0.008). Similar associations were found for FVC-PGS and mean sBP. A significant reduction in sBP was observed per 1-SD increase in FEV_1_ and FVC-PGS **(**Table 2**).** After further adjustments for Apo-A1 and Apo-B the results remained largely unchanged **(Supplement** Table 2**).**

**Table 2:** Univariate regression for mean systolic blood pressure by quartiles of PGS (n = 27,438)

FEV_1_ score	Q1 (highest) (n = 6859)	Q2 (n = 6860)	Q3 (n = 6860)	Q4 (lowest) (n = 6859)	p-value §	Per 1 SD increase (mmHg)
Model 1	140.5 (140.0–141.0)	141.5 (141.0–142.0)	141.2 (140.8–141.7)	141.4 (141.0–141.9)	0.005	−0.389**
Model 2	140.7 (140.3–141.1)	141.4 (141.0–141.8)	141.2 (140.8–141.6)	141.5 (141.1–141.9)	0.008	−0.332**
**FVC score**	**Q1 (highest)** **(n = 6859)**	**Q2** **(n = 6860)**	**Q3 (n = 6860)**	**Q4** **(n = 6859)**	**p-value**	**Per 1 SD increase**
Model 1	140.5 (140.0–141.0)	141.4 (140.9–141.9)	141.1 (140.6–141.5)	141.8 (141.3–142.2)	< 0.001	−0.455***
Model 2	140.7 (140.3–141.1)	141.3 (140.9–141.7)	141.0 (140.6–141.4)	141.7 (141.3–142.1)	0.001	−0.352**

Model 1 unadjusted, Model 2 controlling for age, sex, height, weight, smoking, prevalent diabetes, inhaled corticosteroid use and principal components 1–5 of population structures. § p-value Q1 vs. Q4. P < 0.05 * P < 0.01 ** P < 0.001*** Quartile cut off points for FEV1 score: Q1=−0.32 to 0.61, Q2=−0.49 to −0.32, Q3=−0.65 to −0.49, Q4=−2.52 to −0.65. Quartile cut off point for FVC score: Q1=−0.27 to 0.53, Q2=−0.42 to −0.27, Q3=−0.57 to −0.42, Q4=−1.36 to −0.57

### Health outcomes by quartiles of PGS

**Fig. 2:**
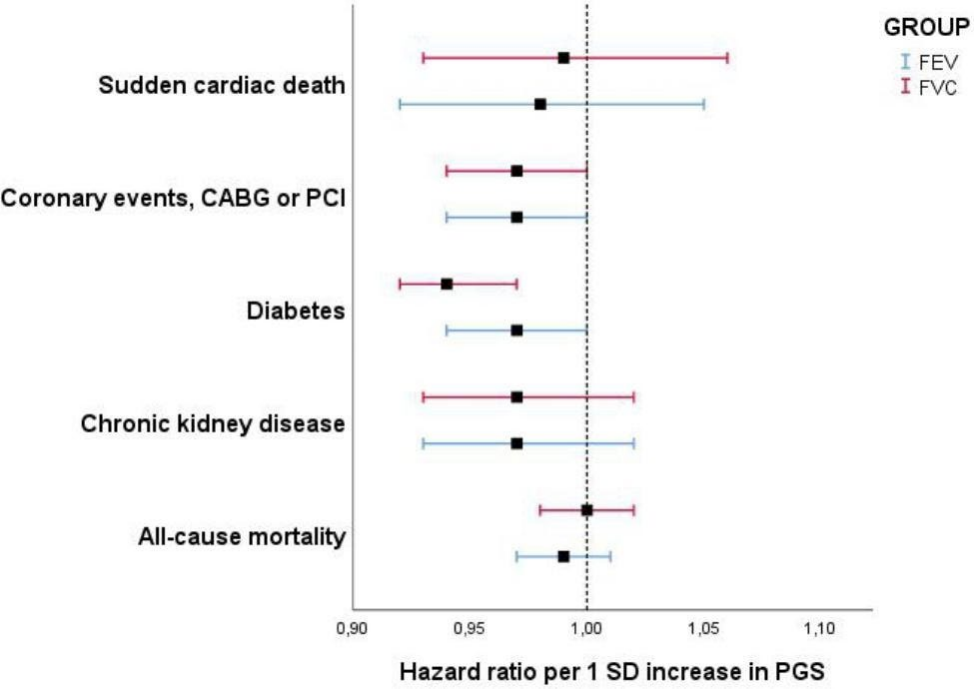
Hazard ratios (95% CI) of outcomes by 1 SD increase in PGS

HR of cardio-metabolic, renal and mortality outcomes by quartiles of and 1-SD increase in FVC and FEV_1_-PGS are shown in Table 3 and Supplement Table 3, respectively and Fig. 2. A low FVC-PGS was significantly associated with the risk of future diabetic events (Model 2, Q1 vs. Q4 HR: 1.22 (1.12–1.32), p-trend < 0.001 (significant at Bonferroni adjusted p-value), HR per 1-SD increase in FVC-PGS 0.94 (0.92–0.97) p-value < 0.001). Although this association was also found for FEV_1_-PGS, the results for FEV1-PGS quartiles were not significant at the Bonferroni adjusted p-value (p-value = 0.015) and borderline significant per 1-SD increase in FEV_1_-PGS (p = 0.04). A low FEV_1_-PGS was however significantly associated with future MACE events (Model 2 Q1 vs. Q4 HR: 1.13 (1.04–1.22), p-trend 0.008, HR per 1-SD increase in FEV_1_-PGS 0.97 (0.94-1.00), p < 0.05). No significant association was observed between low FVC or FEV_1_-PGS and SCD, CKD or all-cause mortality. The results for HR of outcomes by FEV_1_-PGS and FVC-PGS after further adjusting model 2 for Apo-A1 and Apo-B are shown in Supplement Table 4. The results were found to remain largely the same, with a small increase in significance for the p-values related to the MACE outcomes.

As we found that higher education was significantly associated with PGS quartiles in the baseline analysis (Table 1and Supplement Table 1) we additionally added higher education level to the adjustments in Supplement Table 4 and found that results again remained largely unchanged. A 1-SD increase in the FEV_1_-PGS was no longer associated with diabetes after adjusting for higher education, however this association was very borderline significant previously (p = 0.046) and the conclusion that FVC-PGS are more strongly associated with diabetes than FEV_1_-PGS with diabetes remained. The association between sBP and PGS became slightly attenuated after adjusting for higher education however the associations for both quartiles (p-value for Q1 vs. Q4 for FEV_1_-PGS = 0.018 and for FVC-PGS = 0.003) and per 1-SD increase (change in sBP per 1-SD increase in FEV_1_-PGS: -0.289, p-value = 0.008, and per 1-SD increase in FVC-PGS: -0.297, p-value = 0.006) remain statistically significant.

**Table 3:** Health outcomes by quartiles of FVC-PGS and by 1-SD increase in FVC-PGS

Outcome	Model	Q1 (highest)	Q2	Q3	Q4 (lowest)	p-trend	HR per 1 SD increase
SCD events (n = 994)	Model 1	1.00 (ref)	1.04 (0.87–1.24)	0.98 (0.82–1.17)	1.06 (0.89–1.26)	0.696	0.97 (0.91–1.03)
	Model 2	1.00 (ref)	1.00 (0.84–1.19)	0.95 (0.79–1.14)	1.00 (0.84–1.19)	0.850	0.99 (0.93–1.06)
MACE (n = 4640)	Model 1	1.00 (ref)	1.07 (0.98–1.16)	1.09 (1.00-1.18)	1.13 (1.04–1.23)	0.003†	0.96 (0.93–0.99)**
	Model 2	1.00 (ref)	1.04 (0.96–1.13)	1.07 (0.98–1.16)	1.10 (1.01–1.19)	0.022	0.97 (0.94-1.00)
Diabetes events (n = 4613)§	Model 1	1.00 (ref)	1.13 (1.04–1.23)	1.20 (1.10–1.30)	1.28 (1.18–1.39)	< 0.001†	0.92 (0.90–0.95)***
	Model 2	1.00 (ref)	1.10 (1.01–1.20)	1.15 (1.06–1.25)	1.22 (1.12–1.32)	< 0.001†	0.94 (0.92–0.97)***
CKD events (n = 1775)	Model 1	1.00 (ref)	0.97 (0.85–1.11)	1.15 (1.01–1.31)	1.10 (0.96–1.25)	0.035	0.95 (0.90–0.99)*
	Model 2	1.00 (ref)	0.92 (0.80–1.05)	1.10 (0.97–1.26)	1.01 (0.89–1.16)	0.298	0.97 (0.93–1.02)
All-cause mortality (n = 12,126)	Model 1	1.00 (ref)	1.02 (0.97–1.07)	1.02 (0.97–1.08)	1.02 (0.97–1.08)	0.377	0.99 (0.97–1.01)
	Model 2	1.00 (ref)	0.98 (0.94–1.03)	1.00 (0.95–1.05)	0.99 (0.94–1.04)	0.736	1.00 (0.98–1.02)

Model 1: Unadjusted

Model 2: controlling for age, sex, height, weight, smoking, systolic BP, prevalent diabetes and principal components 1–5 of population structures

§ Diabetes outcome not adjusted for prevalent diabetes (no prevalent diabetic events included) and additionally adjusted for inhaled corticosteroid use

MACE: Major adverse Cardiovascular Events (includes coronary events, PCI and CABG)

Population for SCD outcome: 26,915, MACE outcome: 27,164, diabetes: 26,211, CKD: 27,436, Death: 27,438

P < 0.05 * P < 0.01 ** P < 0.001***.

† significant Bonferroni adjusted p-trend (< 0.01).

### Added value of PGS to prediction models for incident MACE and Incident diabetes

An added value of FVC-PGS was observed in predicting diabetes measured by both the NRI and LR test (Table 4). For the category free NRI, more subjects with diabetes events were correctly reclassified as diabetes events than as non-diabetic events after the addition of FVC-PGS (Z-score) to the prediction model. However, the addition of FVC-PGS mainly reclassified non-events of diabetes, where more subjects without diabetes events were correctly reclassified as not having a diabetes events after the addition of FVC-PGS to the prediction model. Similarly, the addition of FVC-PGS improved the fit of the model for prediction of diabetes (LR test p-value < 0.001). There was no significant NRI after the addition of FEV_1_-PGS to prediction models for diabetes, however LR test showed a marginal improvement in model fit (p-value 0.0308). There was no significant NRI or improved goodness of fit for MACE prediction after adding PGS to the prediction models.

**Table 4:** Category free Net Reclassification Index and likelihood ratio test for addition of PGS to prediction model for incident MACE (n = 22,065) and incident diabetes (n = 25,655)

	Category free Net Reclassification Index (NRI)		Likelihood ratio (LR) test	
	**NRI**	**p-value**	**LR chi** ^**2**^ **test**	**p-value**
Incident MACE, addition of FEV_1−_PGS	0.10	0.995	2.21	0.1371
Incident MACE, addition of FVC-PGS	0.46	0.788	1.77	0.1837
Incident Diabetes, addition of FEV_1_-PGS	1.27	0.396	4.67	0.0308
Incident Diabetes, addition of FVC-PGS	4.77	**0.001**	18.07	**< 0.001**

## Discussion

To our knowledge, the present study is the first PGS study for lung function that has assessed a range of different health outcomes beyond COPD, along with its added benefit in risk prediction models. We show that PGS of low lung function is associated with higher sBP at baseline and increased future risk of diabetes in a cohort of middle-aged subjects. There is also a strong added benefit of FVC-PGS in risk prediction models for the outcome of future diabetes. The finding is more strongly observed for low FVC-PGS than FEV_1_-PGS, re-enforcing previous knowledge that highlights the importance of vital capacity of the lung on health.

Studies of PGS for different health outcomes have recently generated considerable interest [30]. Identification of high-risk groups using PGS can be thought of as the equivalent to having additional risk factor for an outcome that could be used in the clinical setting for risk stratification. Additionally, as we also found, PGS can improve risk prediction of certain health outcomes. Tikkanen et al. found that combining traditional risk factors for CAD with a PGS improved risk prediction and risk discrimination for CAD [31]. Previous studies of lung function PGS have mainly assessed respiratory outcomes, predominantly COPD. Lung function PGS can identify subjects at significantly increased risk of COPD and shows improved prediction for COPD when combined with clinical risk factors [12]. We believe we are the first study assessing a broad range of outcomes outside of pulmonary disease for PGS for lung function.

We found a small but significant increase in sBP with lower lung function PGS. The association between BP and lung function has previously found in observational studies [32, 33]. It has been suggested that hypertension in combination with the use of antihypertensive medication and not high BP itself is associated with lower lung function in the general population [34]. Our findings suggest that genetic susceptibility for reduced lung function has a small but significant effect on sBP. However, as BP is so widely affected by objective and subjective factors, the risk of higher sBP associated with genetic susceptibility for lower levels of lung function may be part of variety of other factors influencing the observed measurement. Further studies assessing long-term changes in sBP associated with genetic susceptibility for reduced lung function may help clarify this relationship further.

The significant association found between FVC-PGS and diabetes provides additional evidence of causality, given that causal effect for lung function on diabetes was observed by previous MR analyses [18, 35]. Lung function has been known to be associated with diabetes where adults with diabetes are known to have lower FVC and FEV_1_ than non-diabetic adults. The direction of association and mechanisms had remained unclear in the past [36], however there is now emerging evidence for a bi-directional causal association between lung function and diabetes [35]. An association between low vital capacity as a predictor of diabetes has been consistently found in previous observational studies [5,37–39]. The possible explanations for this link have included reduced physical activity due to poor lung function leading to central obesity, hypoxaemia induced insulin resistance, adverse foetal or early-life conditions that effect organogenesis and metabolic pathway programming leading to insulin resistance and inflammation [37]. We have previously found low lung function to be a significant predictor of diabetes which was not fully explained by obesity, inflammation or smoking [5], concluding that common genetic determinants for low lung function and diabetes could be a potential area for further investigation. The current study adds strong evidence to suggest that genetic susceptibility for reduced lung function can predict the development of diabetes and has added value in prediction models.

A significant association was found between MACE and PGS-FEV1 even after further adjustment for apolipoproteins. Previous findings from many observational studies have found low lung function to be repeatedly independently associated with many cardiovascular outcomes [3, 40]. Previous 2-sample MR studies have found higher lung function to potentially protect against CAD and stroke [18]. Results from an MR study in 2018 found a clear inverse relationship between FEV_1_ and CAD but results were less robust for FVC [17]. This is in accordance with our findings where the trend for MACE across quartiles of PGS was stronger for FEV_1_ than for FVC-PGS. A proposed potential explanation for this was that residual confounding due to height in previous observational studies may have led to an observed association between FVC and CAD. FVC is measure of the lung capacity whereas FEV_1_ is thought of as a measure of severity of airflow limitation, where the former is thought to be more so affected by height and the latter by smoking [17]. This is, however, in contrast to recent findings from a 2-sample MR study on lung function and cardiovascular disease (CVD)[19]. Higbee et al. [19] found FVC to be independently and causally related to CAD, but not FEV_1_ and it was again proposed that confounding may explain the previous observed associations between FEV_1_ and cardiovascular events. [19].

Previous studies have found that multiple underlying processes are likely to contribute to the association between lung function and mortality [41]. It is likely that many unmeasured confounders could explain the associations between lung function and all-cause mortality in observational studies. Additionally, although low levels of lung function have been associated with CVD mortality and pulmonary disease mortality in observational studies, the associations with other causes of mortality are less well established, which may have affected the observed relationships in our study.

### Limitations

The main limitation of genetic studies of this nature is representation. Currently many biobanks recruit subjects mainly from European ancestry. This was also the case for the GWAS study we used to base our PGS on and the study population for the MDCS. This limits the use of genetic screening tools created from subsequent findings to subjects from specific populations that are represented in the biobank databases. Our results are also therefore limited for use in subjects from European ancestry.

Another limitation is potential horizontal pleiotropic effects when using PGS as instrument in inferring putative causal relationships. However, our PGS were constructed using GWAS on lung function [11] where age, age squared, sex, height, smoking, and population structures were considered. PGS associated factors such as sBP, weight, prevalent DM and the actual height of the participants were also accounted in our cox regression models. However, horizontal pleiotropic effects cannot be ruled out and PGS in estimating causal relationships should be interpreted carefully. A complementary approach such as MR analysis is often needed. Indeed, MR analyses have shown causal effects for low lung function on the risk of diabetes and CAD, which is in line with our findings [18, 35].

Spirometry data from MPP has limitations as only one acceptable measurement was needed, and it was done before the current guidelines (e.g. no nose clips were used) and therefore was prone to measurement errors. However, we observed a significant correlation between PGS and lung function measures. The correlation coefficient is relatively low probably due to a low SNP-based heritability of lung function (< 5%) and in part also explained by some of the measurement issues described.

## Conclusion

Genetic susceptibility for reduced lung function is associated with higher sBP and strongly related to an increased risk of future diabetes and to a lesser extent, future coronary events. Using PGS, high risk groups could be early detected so they can make early lifestyle changes attempting to mitigate that risk.

## Electronic supplementary material

Below is the link to the electronic supplementary material.

Supplementary Material 1: **Supplement Table 1**: Baseline association between risk factors and FEV1-PGS (n=27 438). **Supplement Table 2**: Univariate regression for mean systolic blood pressure by quartiles of PGS further adjusted for apolipoproteins (n=26536). **Supplement Table 3**: Health outcomes by quartiles of FEV1 PGS and by 1 SD increase in FEV1 PGS. **Supplement Table 4**: Sensitivity analysis of outcomes after further adjustment for apolipoproteins by FEV1 and FVC PGS.

## Data Availability

The authors do not own the data underlying this study. The data are owned by Lund University, and the approval for research from this database is obtained through the Malmö Diet and Cancer Study (MDCS) steering committee. The data cannot be made publicly available without ethical approval. Data are available upon request for interested researchers by applying to MDCS steering committee. Please email (Anders.Dahlin@med.lu.se) with requests for the data.

## Abbreviations

MI: myocardial infarction
SCD: sudden cardiac death
PGS: polygenic scores
GWAS: genome wide association studies
MR: mendelian Randomization
COPD: chronic obstructive pulmonary disease
CAD: coronary artery disease
T2DM: type 2 diabetes mellitus
sBP: systolic blood pressure
FEV1: forced expiratory volume in 1 s
FVC: forced vital capacity
MDCS: malmö diet and cancer study
CE: coronary events
CKD: chronic kidney disease
Apo A-1: apolipoprotein A1
Apo B: apolipoprotein B
MPP: malmö preventive project
QC: quality control
MAF: minor allele frequency
PCA: principal component analysis
PRS-CS: PRS-continuous shrinkage
SNP: single-nucleotide polymorphism
ICD: international Classification of Diseases
MACE: major adverse cardiovascular events
CABG: coronary artery bypass graft
PCI: percutaneous coronary intervention
MHR: malmö HbA1c register
NDR: swedish national Diabetes register
ANDIS: all New Diabetics in Scania
PC: principal components
NRI: net reclassification index
BMI: body mass index
HR: hazard ratios
Q: quartile
LR test: log-likelihood ratio test
CVD: cardiovascular disease
