# Supplementary material for: Polygenic scores for low lung function and the future risk of adverse health outcomes
Source: Cardiovasc Diabetol. 2022 Nov 3;21:230. doi: 10.1186/s12933-022-01661-y (PMC9635172; doi:10.1186/s12933-022-01661-y)
Supplement: Supplementary file 1 — Supplementary Material 1: Supplement Table 1: Baseline association between risk factors and FEV1-PGS (n=27 438). Supplement Table 2: Univariate regression for mean systolic blood pressure by quartiles of PGS further adjusted for apolipoproteins (n=26536). Supplement Table 3: Health outcomes by quartiles of FEV1 PGS and by 1 SD increase in FEV1 PGS. Supplement Table 4: Sensitivity analysis of outcomes after further adjustment for apolipoproteins by FEV1 and FVC PGS. [file 12933_2022_1661_MOESM1_ESM.docx]

**Supplement Table 1:** Baseline association between risk factors and FEV_1_-PGS (n=27 438)

|  | **Q1 (highest)**  **-0.32 - 0.61**  **(n=6859)** | **Q2**  **-0.49 - -0.32**  **(n=6860)** | **Q3**  **-0.65 - -0.49**  **(n=6860)** | **Q4 (lowest)**  **-2.52 - -0.65**  **(n=6859)** | **p-value** |
| --- | --- | --- | --- | --- | --- |
| Age (years) | 57.9 (±7.5) | 58.2 (±7.7) | 58.2 (±7.7) | 58.1 (±7.7) | 0.284 |
| Height (cm) | 168.3 (±8.9) | 168.7 (±8.8) | 168.7 (±8.8) | 168.9 (±8.9) | <0.001 |
| BMI (kg/m^2^) | 25.7 (±4.0) | 25.8 (±4.0) | 25.8 (±4.0) | 25.7 (±4.0) | 0.527 |
| Systolic BP (mmHg) | 140.5 (±20.0) | 141.5 (±19.9) | 141.3 (±20.1) | 141.5 (±20.0) | 0.016 |
| Current smokers (%) | 28.4 | 28.0 | 27.9 | 28.7 | 0.103 |
| ApoA1* | 156.7 (±27.4) | 157.0 (±28.6) | 156.9 (±28.4) | 156.6 (28.3) | 0.871 |
| ApoB* | 107.0 (±26.5) | 106.8 (±26.2) | 107.3 (±26.1) | 107.1 (±25.4) | 0.531 |
| Prevalent CE (%) | 1.8 | 1.9 | 2.0 | 1.9 | 0.515 |
| Prevalent DM (%) | 3.8 | 4.3 | 4.6 | 5.2 | <0.001 |
| Inhaled corticosteroid use (%) | 1.9 | 1.8 | 2.3 | 2.4 | 0.011 |
| Higher education (%) | 15.1 | 14.8 | 14.2 | 12.8 | <0.001 |
| Low physical activity (%) ** | 19.7 | 20.4 | 20.5 | 20.1 | 0.501 |

*Data from 26 536 subjects on lipoproteins

** Data from 27237 subjects on physical activity score

**Supplement Table 2:** Univariate regression for mean systolic blood pressure by quartiles of PGS further adjusted for apolipoproteins (n=26536)

| **FEV_1_ score** | Q1 (highest) | Q2 | Q3 | Q4 (lowest) | p-value § | Per 1 SD increase (mmHg) |
| --- | --- | --- | --- | --- | --- | --- |
|  | 140.7 (140.3-141.1) | 141.4 (141.0-141.9) | 141.2 (140.7-141.6) | 141.5 (141.1-141.9) | 0.009 | -0.318** |
| **FVC score** | Q1 (highest) | Q2 | Q3 | Q4 | p-value | Per 1 SD increase |
|  | 140.8 (140.3-141.1) | 141.4 (140.9-141.8) | 141.0 (140.6-141.4) | 141.7 (141.3-142.1) | 0.002 | -0.318** |

Adjustments: age, sex, height, BMI, smoking, prevalent diabetes, Apolipoprotein A1 (Apo A-1) and Apolipoprotein B (Apo B), inhaled corticosteroid use and principal components 1-5 of population structures. § p-value Q1 vs Q4. P<0.05 * P <0.01 ** P<0.001*** Quartile cut off points for FEV1 score: Q1=-0.32 – 0.61, Q2=-0.49 - -0.32, Q3=-0.65 - -0.49, Q4=-2.52 - -0.65. Quartile cut off point for FVC score: Q1=-0.27 – 0.53, Q2=-0.42 - -0.27, Q3=-0.57 - -0.42, Q4=-1.36 - -0.57

**Supplement Table 3:** Health outcomes by quartiles of FEV_1_ PGS and by 1 SD increase in FEV_1_ PGS

| Outcome | Model | Q1  (highest) | Q2 | Q3 | Q4  (lowest) | p-trend | Per 1 SD increase |
| --- | --- | --- | --- | --- | --- | --- | --- |
| SCD events  (n=994) | Model 1 | 1.00 (ref) | 1.24 (1.04-1.47) | 1.03 (0.86-1.24) | 1.17 (0.98-1.40) | 0.335 | 0.97 (0.91-1.03) |
|  | Model 2 | 1.00 (ref) | 1.22 (1.02-1.45) | 1.02 (0.85-1.23) | 1.13 (0.94-1.35) | 0.534 | 0.98 (0.92-1.05) |
| MACE (n=4640) | Model 1 | 1.00 (ref) | 1.07 (0.99-1.17) | 1.06 (0.97-1.15) | 1.15 (1.06-1.25) | 0.003† | 0.96 (0.93-0.99)** |
|  | Model 2 | 1.00 (ref) | 1.06 (0.97-1.15) | 1.05 (0.96-1.14) | 1.13 (1.04-1.22) | 0.008† | 0.97 (0.94-1.00)* |
| Diabetes events§  (n=4613) | Model 1 | 1.00 (ref) | 1.12 (1.03-1.21) | 1.09 (1.00-1.18) | 1.14 (1.05-1.24) | 0.007† | 0.96 (0.94-0.99)* |
|  | Model 2 | 1.00 (ref) | 1.11 (1.02-1.20) | 1.09 (1.00-1.18) | 1.12 (1.03-1.22) | 0.015 | 0.97 (0.94-1.00)* |
| CKD events (n=1775) | Model 1 | 1.00 (ref) | 1.10 (0.96-1.26) | 1.18 (1.03-1.34) | 1.13 (0.99-1.29) | 0.044 | 0.96 (0.91-1.00) |
|  | Model 2 | 1.00 (ref) | 1.08 (0.94-1.23) | 1.15 (1.01-1.31) | 1.09 (0.95-1.25) | 0.135 | 0.97 (0.93-1.02) |
| All-cause mortality (n=12126) | Model 1 | 1.00 (ref) | 1.04 (0.99-1.09) | 1.02 (0.97-1.07) | 1.06 (1.00-1.11) | 0.082 | 0.98 (0.96-1.00)* |
|  | Model 2 | 1.00 (ref) | 1.01 (0.96-1.07) | 1.00 (0.95-1.05) | 1.04 (0.99-1.09) | 0.266 | 0.99 (0.97-1.01) |

Model 1: Unadjusted

Model 2: controlling for age, sex, height, weight, smoking, systolic BP, prevalent diabetes and principal components 1-5 of population structures.

§ Diabetes outcome not adjusted for prevalent diabetes (no prevalent diabetic events included) and additionally adjusted for inhaled corticosteroid use.

MACE: Major adverse Cardiovascular Events (includes coronary events, PCI and CABG)

Population for SCD outcome: 26915, MACE outcome: 27164, diabetes: 26211, CKD: 27436, Death:27438. P<0.05 * P <0.01 ** P<0.001***

† significant Bonferroni adjusted p-trend (<0.01)

**Supplement Table 4:** Sensitivity analysis of outcomes after further adjustment for apolipoproteins by FEV_1_ and FVC PGS

| Outcome |  | Q1  (highest) | Q2 | Q3 | Q4  (lowest) | p-trend | HR per 1 SD increase |
| --- | --- | --- | --- | --- | --- | --- | --- |
| SCD events  (n=952) | FEV_1_-PGS | 1.00  (ref) | 1.22 (1.02-1.46) | 1.03 (0.85-1.24) | 1.15 (0.95-1.38) | 0.445 | 0.98 (0.92-1.05) |
|  | FVC-PGS | 1.00 (ref) | 1.01 (0.84-1.21) | 0.95 (0.79-1.14) | 1.01 (0.84-1.21) | 0.920 | 0.99 (0.93-1.06) |
| MACE  (n=4476) | FEV_1_-PGS | 1.00 (ref) | 1.08 (0.99-1.17) | 1.05 (0.96-1.14) | 1.15 (1.06-1.25) | 0.003† | 0.96 (0.93-0.99)** |
|  | FVC-PGS | 1.00 (ref) | 1.04 (0.96-1.14) | 1.08 (0.99-1.17) | 1.11 (1.02-1.21) | 0.011 | 0.97 (0.94-1.00)* |
| Diabetes events  (n=4426) | FEV_1_-PGS | 1.00 (ref) | 1.12 (1.03-1.22) | 1.07(0.99-1.17) | 1.13 (1.04-1.23) | 0.021 | 0.97 (0.94-1.00)* |
|  | FVC-PGS | 1.00 (ref) | 1.10 (1.01-1.20) | 1.15 (1.05-1.25) | 1.22 (1.12-1.32) | <0.001† | 0.94 (0.92-0.97)*** |
| CKD events  (n=1716) | FEV_1_-PGS | 1.00 (ref) | 1.07 (0.94-1.23) | 1.18 (1.03-1.35) | 1.09 (0.95-1.25) | 0.118 | 0.97 (0.93-1.02) |
|  | FVC-PGS | 1.00 (ref) | 0.92 (0.80-1.06) | 1.11 (0.97-1.27) | 1.02 (0.89-1.17) | 0.240 | 0.97 (0.93-1.02) |
| All-cause mortality  (n=11696) | FEV_1_-PGS | 1.00 (ref) | 1.02 (0.97-1.07) | 1.00 (0.95-1.06) | 1.04 (0.99-1.10) | 0.202 | 0.99 (0.97-1.00) |
|  | FVC-PGS | 1.00 (ref) | 0.99 (0.94-1.04) | 1.00 (0.95-1.06) | 0.99 (0.94-1.04) | 0.785 | 1.00 (0.98-1.02) |

Adjustments: age, sex, height, weight, smoking, systolic BP, prevalent diabetes, Apolipoprotein A1 (Apo A-1) and Apolipoprotein B (Apo B) and principal components 1-5 of population structures. § Diabetes outcome not adjusted for prevalent diabetes (no prevalent diabetic events included) and additionally adjusted for inhaled corticosteroid use. MACE: Major adverse Cardiovascular Events (includes coronary events, PCI and CABG)

Population for SCD outcome: 26035, MACE outcome: 26273, diabetes: 25376, CKD:26534, Death: 26536. P<0.05 * P <0.01 ** P<0.001*** † significant Bonferroni adjusted p-trend (<0.01)

Regeneron Genetics Center Banner Author List and Contribution Statements

**RGC Management and Leadership Team**

Goncalo Abecasis, D.Phil. , Aris Baras, M.D. , Michael Cantor, M.D. , Giovanni Coppola, M.D. , Andrew Deubler , Aris Economides, Ph.D. , Katia Karalis, Ph.D. , Luca A. Lotta, M.D., Ph.D. , John D. Overton, Ph.D. , Jeffrey G. Reid, Ph.D. , Katherine Siminovitch, M.D. , Alan Shuldiner, M.D.

**Sequencing and Lab Operations**

Christina Beechert , Caitlin Forsythe, M.S. , Erin D. Fuller , Zhenhua Gu, M.S. , Michael Lattari , Alexander Lopez, M.S. , John D. Overton, Ph.D. , Maria Sotiropoulos Padilla, M.S. , Manasi Pradhan, M.S. , Kia Manoochehri, B.S. , Thomas D. Schleicher, M.S. , Louis Widom , Sarah E. Wolf, M.S.

**Clinical Informatics**

Amelia Averitt, Ph.D. , Nilanjana Banerjee, Ph.D. , Michael Cantor, M.D. , Dadong Li, Ph.D. , Sameer Malhotra, M.D. , Deepika Sharma, MHI , Jeffrey Staples , Ph.D.

**Genome Informatics**

Xiaodong Bai, Ph.D. , Suganthi Balasubramanian, Ph.D. , Suying Bao, Ph.D. , Boris Boutkov, Ph.D. , Siying Chen, Ph.D. , Gisu Eom, B.S. , Lukas Habegger, Ph.D. , Alicia Hawes, B.S. , Shareef Khalid , Olga Krasheninina, M.S. , Rouel Lanche, B.S. , Adam J. Mansfield, B.A. , Evan K. Maxwell, Ph.D. , George Mitra, B.A. , Mona Nafde, M.S. , Sean O’Keeffe, Ph.D. , Max Orelus, B.B.A. , Razvan Panea, Ph.D. , Tommy Polanco, B.A. , Ayesha Rasool, M.S. , Jeffrey G. Reid, Ph.D. , William Salerno, Ph.D. , Jeffrey C. Staples, Ph.D. , Kathie Sun, Ph.D.

**Analytical Genomics and Data Science**

Goncalo Abecasis, D.Phil. , Joshua Backman, Ph.D. , Amy Damask, Ph.D. , Lee Dobbyn, Ph.D. , Manuel Allen Revez Ferreira, Ph.D. , Arkopravo Ghosh, M.S. , Christopher Gillies, Ph.D. , Lauren Gurski, B.S. , Eric Jorgenson, Ph.D. , Hyun Min Kang, Ph.D. , Michael Kessler, Ph.D. , Jack Kosmicki, Ph.D. , Alexander Li , Ph.D. , Nan Lin, Ph.D. , Daren Liu, M.S. , Adam Locke, Ph.D. , Jonathan Marchini, Ph.D. , Anthony Marcketta, M.S. , Joelle Mbatchou, Ph.D. , Arden Moscati, Ph.D. , Charles Paulding, Ph.D. , Carlo Sidore, Ph.D. , Eli Stahl, Ph.D. , Kyoko Watanabe, Ph.D. , Bin Ye, Ph.D. , Blair Zhang, Ph.D. , Andrey Ziyatdinov, Ph.D.

**Therapeutic Area Genetics**

Ariane Ayer, B.S. , Aysegul Guvenek, Ph.D. , George Hindy, Ph.D. , Giovanni Coppola, M.D. , Jan Freudenberg, M.D. , Jonas Bovijn M.D. , Katherine Siminovitch, M.D. , Kavita Praveen, Ph.D. , Luca A. Lotta, M.D. , Manav Kapoor, Ph.D. , Mary Haas, Ph.D. , Moeen Riaz , Ph.D. , Niek Verweij, Ph.D. , Olukayode Sosina, Ph.D. , Parsa Akbari, Ph.D. , Priyanka Nakka, Ph.D. , Sahar Gelfman, Ph.D. , Sujit Gokhale, B.E. , Tanima De, Ph.D. , Veera Rajagopal, Ph.D. , Alan Shuldiner, M.D. , Bin Ye, Ph.D. , Gannie Tzoneva, Ph.D. , Juan Rodriguez-Flores, Ph.D.

**RGC Biology**

Shek Man Chim, Ph.D. , Valerio Donato, Ph.D. , Aris Economides, Ph.D. , Daniel Fernandez, M.S. , Giusy Della Gatta, Ph.D. , Alessandro Di Gioia, Ph.D. , Kristen Howell, M.S. , Katia Karalis, Ph.D. , Lori Khrimian, Ph.D. , Minhee Kim, Ph.D. , Hector Martinez , Lawrence Miloscio, B.S. , Sheilyn Nunez, B.S. , Elias Pavlopoulos, Ph.D. , Trikaldarshi Persaud, B.S.

**Research Program Management & Strategic Initiatives**

Esteban Chen, M.S. , Marcus B. Jones, Ph.D. , Michelle G. LeBlanc, Ph.D. , Jason Mighty, Ph.D. , Lyndon J. Mitnaul, Ph.D. , Nirupama Nishtala, Ph.D. , Nadia Rana, Ph.D.
